# Transition across a sharp interface: Data from Raman and Brillouin imaging spectroscopy

**DOI:** 10.1016/j.dib.2020.106368

**Published:** 2020-10-02

**Authors:** Silvia Caponi, Daniele Fioretto, Maurizio Mattarelli

**Affiliations:** a Istituto Officina dei Materiali del CNR (CNR-IOM) Unità di Perugia, c/o Dip. di Fisica e Geologia, Università di Perugia, Perugia, Italy; b Dipartimento di Fisica e Geologia, Università di Perugia, Via A. Pascoli, Perugia I-06100, Italy

**Keywords:** Brillouin spectroscopy, Raman spectroscopy, Phonons, Interface

## Abstract

Brillouin and Raman imaging are powerful techniques for the investigation of complex materials and they are widely used in material science and biophysics [1], [2], [3], [4], [5], [6], [7]. When dealing with microstructures, the results interpretation requires an accurate understanding of the interaction processes in presence of acoustic and chemical boundaries between different materials [8], [9], [10], [11], [12], [13], [14], [15]. The data here reported are obtained while scanning with sub-micron resolution the sharp interfaces between vitreous-SiO_2_/Water and Polyethylene (PET)/Glycerol. Molecular and acoustic vibrations were observed by means of a recently developed micro-spectrometer, which acquires simultaneously Raman and Brillouin spectra on the same point with high spatial and spectral resolution [3]. Two external optic configurations were adopted in order to evidence the dependency of the measurements on the optical scattering volume. The evolution of the detected phonon modes, propagating and not propagating, is obtained by a direct observation of the raw data for the two interfaces, which present different acoustic mismatch. These experimental records can be exploited by researchers employing Raman and Brillouin imaging to discuss the resolution limit of the techniques and to compare the effect of different experimental set-ups. Moreover, thanks to their high spectral resolution they can be useful to researchers working on acoustic phonon transport at interfaces to model the dependency of transmission of long wavelength phonons on the acoustic mismatch.

## Specifications Table

**Table:** 

Subject	Material Characterization
Specific subject area	Optical Brillouin and Raman spectroscopy
Type of data	Raw and graph
How data were acquired	Custom-built Microscope coupled with Brillouin and Raman spectrometers [3,7]
Data format	Raw
Parameters for data collection	The laser beam with λ=532 nm and power on the sample P∼10 mW is focalized using two different objectives: Mitutoyo M Plan Apo 20x with NA 0.42 and Olympus UPLSAPO 60XW with NA 1.2. The focus point of the laser beam was shifted across the investigated interface by a xyz translator stage (PI 611–3S Nanocube XYZ). The stage is characterized by a long travel (100 µm) and a high spatial resolution (10 nm) granted by a piezoelectric control [7].
Description of data collection	The Raman spectra were acquired by RM- Horiba iHR320 Triax using a 600 grooves/mm grating and a liquid N_2_ cooled CCD detector (1024 × 256 pixels). This allows the acquisition of Raman spectra in the range 150–3800 cm^−1^ frequency shift. The Brillouin spectra were acquired simultaneously, by means of the high contrast tandem interferometer TFP-2 from JRS Scientific.
Data source location	Institution: Department of Physics and Geology, University of Perugia. City: Perugia Country: Italy
Data accessibility	With the article
Related research article	S. Caponi, D. Fioretto, M. Mattarelli, On the actual spatial resolution of Brillouin Imaging, Opt. Lett. 45 (2020) 1063. https://doi.org/10.1364/ol.385072.

## Value of the Data

• The acquired data present a correlative characterization of the transitions of chemical and mechanical properties when crossing the interface between materials as measured by Brillouin-Raman micro-spectroscopy. They provide insight into the propagation of phonons across an interface.
• These data can be exploited by researchers employing Raman and Brillouin imaging to discuss the resolution limit of the techniques and to compare the effect of different experimental set-ups. Moreover, they can be useful to researchers working on acoustic phonon transport at interfaces.
• Thanks to the high resolution and contrast of the Brillouin experimental set-up, the data present a high spectral quality and offer the possibility to test different models for addressing the changes of the measured phonon properties across an interface.
• They can be used to expand the database of characterized material interfaces with different acoustic mismatch for developing a comprehensive theoretical framework.

## 1 Data Description

The shared data were recorded investigating two different interfaces (SiO_2_/water and PET/Glycerol) using a combined Brillouin and Raman spectrometer recently built up in our Lab [3] . The whole dataset is archived in “BRInt_Dataset.zip” attached in "Appendix A. Supplementary data" of the present article. Both Raman and Brillouin data are simultaneously acquired probing the same point of the samples. They were collected as a function of position during a lateral scan perpendicular to the interface, having fixed the focal plane on the upper surface of the SiO_2_ or PET slab. The data are divided in a single folder per interface and external optics (60X or 20X). In turn, for each experimental configuration there are two folders with the coupled Brillouin and Raman spectra in ascii format. The progressive number at the end of each filename represents the position of measurement.

**Fig. 1:**
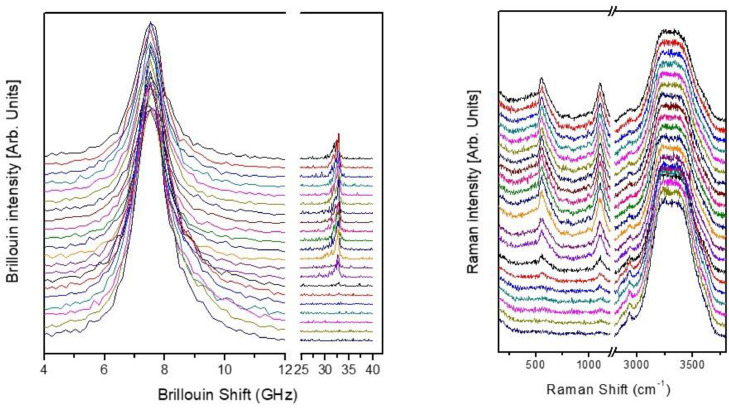
Sequence of Brillouin (left) and Raman (right) spectra acquired with the 60X objective across the SiO_2_/water interface. The measurements are performed without modifying the experimental configuration but by moving the scattering volume perpendicular to the interface in steps of 0.3 µm.

Fig. 1 shows the Brillouin (left) and Raman (right) spectra acquired across the SiO_2_/water interface for a scan consisting of 6 µm as a whole. The Brillouin spectra have been hidden between 12 and 25 GHz as no peak is present in that region. Likewise, the Raman spectra only shows the regions of the main peaks of water and SiO_2_. However, the raw data in the attached zip archive present the complete acquired data range. The spectra have been normalized to the peak related with water (∼7.7 GHz for Brillouin spectra and the band centred at 3500 cm^−1^ for Raman spectra) In the spectra there is a clear evolution of the intensity of modes corresponding to the SiO2 as the scattering volume is moved across the interface.

**Fig. 2:**
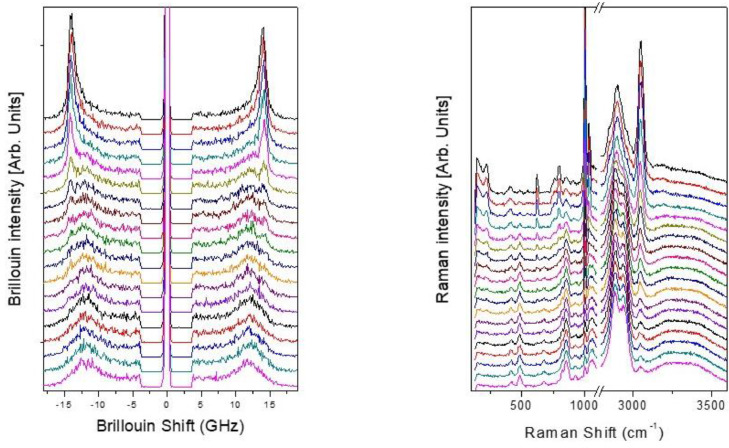
Sequence of Brillouin (left) and Raman (right) spectra acquired with the 60X objective across the PET/Glycerol interface. The measurements are performed without modifying the experimental configuration but by moving the scattering volume perpendicular to the interface in steps of 0.5 µm.

Fig. 2 shows the Brillouin (left) and Raman (right) spectra acquired across the PET/Glycerol interface for a scan consisting of 9 µm as a whole. The Raman spectra shows the region between about 100 and 1100 cm^−1^, where the skeletal vibrations of PET and glycerol are present, and the region between 2800 and 3600 cm^−1^, which contains the stretching peaks of CH and OH. Both in the Raman and Brillouin spectra there is an evolution with a change of the relative intensity of the bands related with Glycerol and PET.

In both Fig. 1 and Fig. 2, the spectra were shifted along the ordinate axis to improve readability. No background was removed from either Brillouin or Raman spectra.

## 2 Experimental Design, Materials and Methods

The measurements were performed by a Custom-built microscope combined with Raman and Brillouin spectrometers [3]. The Raman spectrometer (Horiba iHR320 Triax using a 600 grooves/mm grating and a N2 cooled CCD detector) analysed the Stokes region of the backscattered VV polarized signal in the range between 125 and 3800 cm−1 The actual spectral resolution of the Raman measurements was about 10 cm^−1^, corresponding to 3 pixels. The Brillouin spectrometer is the High Contrast HC version of the Sandercock type tandem Fabry Perot (TFP-2) interferometer. In the measurement configuration, the free spectral range was 43 GHz when testing the SiO_2_/water interface and 30 GHz for the PET/Glycerol one. The actual spectral resolution is about 0.4 and 0.3 GHz, respectively. The excitation was provided by a DPSS laser operating at 532 nm. The power on the samples was about 10 mW. The measurements were carried on in back-scattering configuration, with the same microscope objective focalizing the exciting laser into the sample and collecting the scattered light in VV polarization. Two different focussing optics were adopted: A Mitutoyo M Plan Apo 20x objective with NA 0.42 and Olympus UPLSAPO 60XW objective with NA 1.2, operating in water. The lateral resolution was 0.9 and 0.3 µm, respectively.

The SiO_2_ and PET slabs were commercial substrate 1 mm and 0.5 mm thick, respectively, which were snapped after a preliminary cut by a point diamond tool. PET sample was brought to low temperature before snapping to produce a sharper interface and to decrease the residual stresses which can modify the observed phonon properties[14]. The samples were fixed to a Petri dish, which was later filled with reagent grade glycerol or distilled water. For the measurements with the 60XW objective the PET samples were covered by a microscope cover slips, in turn wetted by distilled water. During measurements, the focal plane was positioned on the upper surface of the SiO2 or PET slab. The sample was moved by a xyz translator stage (PI 611–3S Nanocube XYZ), reaching a spatial resolution of 10 nm in a motion range of 100 μm for each axis. The scan was performed by a lateral translation perpendicular to the interface with a step of 0.5 µm, apart from the 60X measurement of the SiO_2_/water interface for which it was 0.3 μm. The duration of measurements was between 2 and 3 s per Raman spectrum and between 2.6 and 26 s per Brillouin spectrum (the actual values are reported in the attached dataset). All measurements were carried out at room temperature.

## Declaration of Competing Interest

The authors declare that they have no known competing financial interests or personal relationships which have or could be perceived to have influenced the work reported in this article.

## Appendix Supplementary materials

Image, application 1
